# A mathematical model to understand the mechanisms of action of class 1 antiarrhythmic drugs

**DOI:** 10.4103/0253-7613.66848

**Published:** 2010-06

**Authors:** Dinesh K. Jain, Raj K. Arya, Ashok K. Jain

**Affiliations:** Department of Pharmacology, G.R. Medical College, Gwalior, Madhya Pradesh - 474 001, India. E-mail: dkumjain@hotmail.com

Sir,

The pharmacology of antiarrhythmic drugs is very difficult to understand, specially that of class 1 antiarrhythmic drugs. One drug, quinidine, of group Ia increases the action potential duration (APD) and effective refractory period (ERP) and slows repolarization by blocking Na channels. A second drug, lignocaine, of group Ib, with the same Na-channel blocking action, decreases ERP and APD and accelerates repolarization, while a third drug, flecainide, of group Ic, with the same Na-channel blocking action alters APD and ERP and alters repolarization minimally or does not alter it at all.[1] When students enquire about the mechanism for these variations, it becomes very difficult to explain.

The action potential (AP) in Purkinje fibers can be divided into follwing phases: rapid depolarization phase O, early repolarization phase 1, plateau phase 2, rapid repolarization phase 3 and resting phase, called as slow diastolic depolarization.

**Figure 1:**
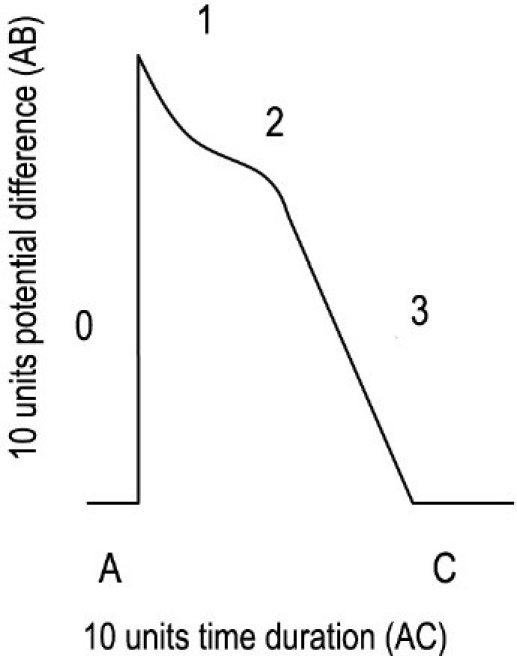
Normal action potential duration of 10 units time (A to C).

Phase 0 (phase of rapid depolarization) is due to the entry of Na ions through fast inward Na^+^ channels. The cell membrane's electrical charge changes from negative (-90 mv) to zero and finally to positive +30 mv. Transient outward K^+^ current (I_to_) opens briefly immediately after depolarization and contributes to early repolarization (phase 1) and outward (delayed) rectifying K^+^ current (I_K_) opens in phase 2 (plateau) and initiates repolarization[2] [Figure 1]

Quinidine, procainamide (Ia) and flecainide (I_C_ ) block K^+^ channels while lignocaine (Ib) does not block K^+^ channels during repolarization. The class Ia drugs – quinidine and procainamide – block I_K_ - and I _to_ -potassium channels, the class Ib drug – lignocaine – does not block any K ^+^ channel while the class Ic drugs – flecainide and encainide – block I_K_ -potassium channels only. Class Ic drugs do not block I_to_ -potassium channels.[3] “ERP is closely related to the APD.”[4]

In normal AP of Purkinje fibers, the difference of voltage from -90 mv to +30 mv (difference, 120 mv) in 0 phase can be represented by 10 units height from A to B in the diagram. Total time taken from the beginning of 0 phase to the end of phase 3 can be presumed as 10 units time, as represented in the diagram from point A to point C. Ten units height of voltage difference will take 10 units time to return to the resting potential at the end of phase 3. The horizontal axis represents time of completing AP and the vertical axis represents the voltage difference during 0 phase of AP.

Normal voltage difference in the 0 phase of AP in Purkinje fibers is from -90 mv to +30 mv, which is equivalent to 120 mv, and can be represented by 10 units height. We can presume that if a drug blocks the Na^+^ channels mildly in the 0 phase, the height of AP during the 0 phase will be reduced from 10 units to 9 units. If a drug blocks the Na^+^ channels moderately in the 0 phase, the height of AP during the 0 phase will be reduced from the normal 10 units to 8 units. If a drug blocks the Na^+^ channels profoundly, the height of AP during the 0 phase will be reduced from the normal 10 units to 7 units.

We can imagine that 1 unit height during the 0 phase of depolarization will take 1 unit time to complete repolarization or AP. If the height is 7 units, then it will take 7 units time to complete AP. If the height is 9 units, then it will take 9 units time to complete AP.

**Figure 2:**
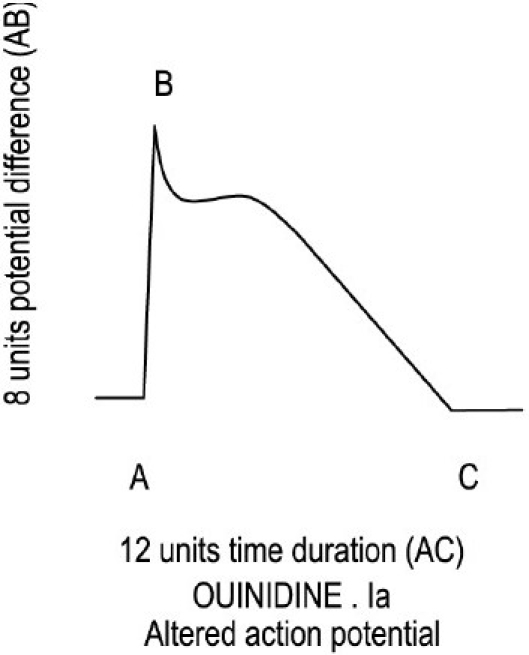
Increased action potential duration by 2 units time (A to C).

Quinidine, a class Ia drug, blocks Na^+^ channels with moderate intensity during the 0 phase of AP. Hence, it reduces the height of the voltage difference from the normal 10 units to 8 units. Quinidine also blocks the I_to_-K^+^ channels during early repolarization of phase 1 and blocks the I_K_-K^+^ channels during phase 2 and phase 3 of AP. Blocking of K^+^ channels during repolarization will prolong the repolarization time. By blocking the I_to_ channels, quinidine prolongs 1 unit time and by blocking the I_K_ channels, quinidine prolongs 3 units time to complete repolarization. If the K^+^ channels are not blocked by quinidine, then the time taken to come at the resting potential would have been 8 units, and quinidine could decrease the APD and ERP. But, quinidine, by the blocking I_to_ and I_K_ channels, prolongs the time of APD equivalent to (8 + 1 + 3) 12 units. The normal time of APD when drug does not block the Na^+^ and K^+^ channels is 10 units. Now, it increases to 12 units. Time to complete AP is APD and ERP. In this way, by this mathematical model, we can understand how quinidine increased the APD and ERP. Quinidine also slows repolarization because it increases the repolarization time from the normal 10 units to 12 units [Figure 2 and Table 1].

**Table 1:** Altered action potential of class-1 antiarrhythmic drugs by mathematical model

*Name of drugs (A)*	*Intensity of Na-channels blocking action during 0 phase (B)*	*Presumed voltage difference during 0 phase (C)*	*Presumed APD if K-channel is not blocked (D)*	*K-channel blocking action during phase 1, phase 2 phase and 3 (E)*	*Increased time of repolarization due to K-channel blocking action (F)*	*Presumed total time of APD (G)*
Normal	Nil	10 units	10 units	Nil	Nil	10 units
Quinidine 1a	Moderate	8 units	8 units	Blocks I_to_ and I_k_	4 units	12 units
Lignocaine 1b	Mild	9 units	9 units	Nil	Nil	9 units
Flecainide 1c	Profound	7 units	7 units	Blocks I_k_	3 units	10 units

Table showing that intensity of the Na-channel blocking action of drugs (B) reduces the potential differences of 0phase accordingly (C), which reduces the action potential duration (APD) time (D) but the K-channel blocking action (E) also increases the repolarization time (F). Then, the total repolarization time can be calculated, which is closely related to the APD (G).

**Figure 3:**
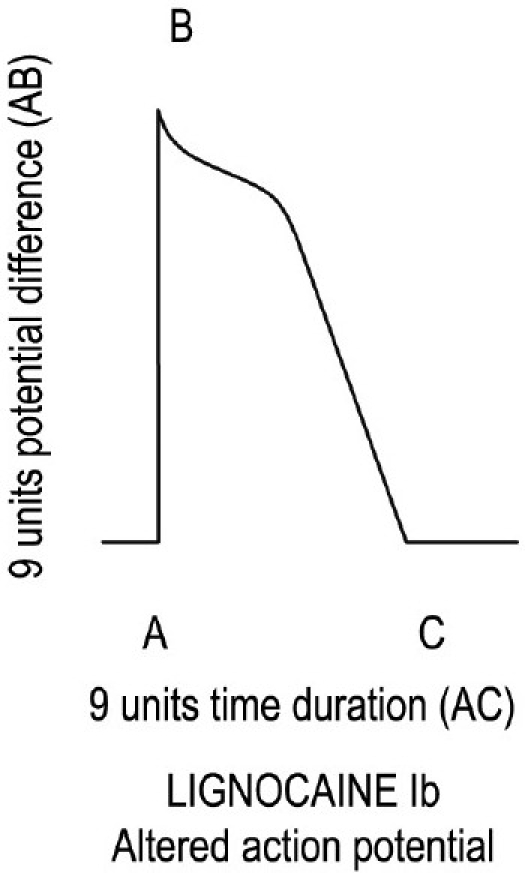
Decreased action potential duration by 1 unit time.

Lignocaine, a class Ib drug, blocks the Na^+^ channels mildly and it can be presumed that it will decrease the height of AP during zero 0 from 10 units to 9 units. Lignocaine does not block the I_to_ and I_K_ channels and that is why it will take 9 units time to come to the resting potential, which is less than 10 units (normal time) to complete APD. Therefore, lignocaine decreases the APD and ERP. Lignocaine slows repolarization because it decreases the repolarization time from the normal 10 units to 9 units [Figure 3 and Table 1].

**Figure 4:**
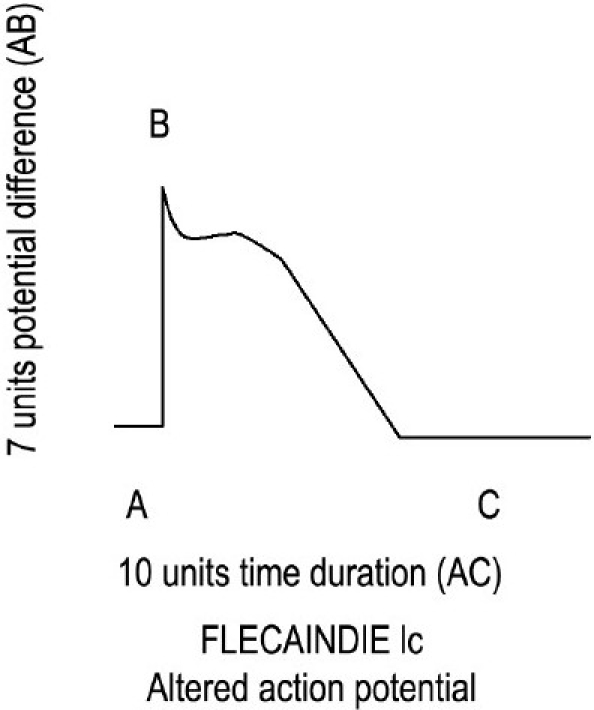
Normal action potential duration.

Flecainide blocks the Na^+^ channels during 0 phase with profound intensity, which will decrease the height of the 0 phase from 10 units to 7 units. Flecainide does not block I_to_ (K^+^ channels) during phase 1 but blocks I_K_ channels during phase 2 and phase 3 of AP. In this way, flecainide increases the time of APD by 3 units. If I_K_ (K^+^ channels of phase 2 and phase 3) is not blocked by flecainide, then time taken by total AP would have been 7 units only. But, by blocking the I_K_ channels, the time of APD increases from 7 units to 10 units. Ten units is the normal time to complete APD when the drug does not block any channel. In this way, we can explain why flecainide does not alter the APD and ERP and the repolarization time [Figure 4 and Table 1].

This mathematical model thus explains how quinidine increases the APD and ERP and slows the repolarization. Lignocaine decreases the APD and ERP and fastens repolarization. Flecainide does not alter the APD and ERP and the repolarization time.

Note: All Na^+^-channel blocker drugs increase the time of 0 phase of AP, but we can ignore it to simplify this model because it is very less in comparison to the total APD.
